# Unsupervised statistical discovery of spaced motifs in prokaryotic genomes

**DOI:** 10.1186/s12864-016-3400-0

**Published:** 2017-01-05

**Authors:** Hao Tong, Paul Schliekelman, Jan Mrázek

**Affiliations:** 1 Department of Statistics, University of Georgia, Athens, GA 30602 USA; 2 Department of Microbiology and Institute of Bioinformatics, University of Georgia, Athens, GA 30602 USA

**Keywords:** Genome, Sequence motifs, Motif-finding, Bacteria, Archaea, DNA sequence repeats

## Abstract

**Background:**

DNA sequences contain repetitive motifs which have various functions in the physiology of the organism. A number of methods have been developed for discovery of such sequence motifs with a primary focus on detection of regulatory motifs and particularly transcription factor binding sites. Most motif-finding methods apply probabilistic models to detect motifs characterized by unusually high number of copies of the motif in the analyzed sequences.

**Results:**

We present a novel method for detection of pairs of motifs separated by spacers of variable nucleotide sequence but conserved length. Unlike existing methods for motif discovery, the motifs themselves are not required to occur at unusually high frequency but only to exhibit a significant preference to occur at a specific distance from each other. In the present implementation of the method, motifs are represented by pentamers and all pairs of pentamers are evaluated for statistically significant preference for a specific distance. An important step of the algorithm eliminates motif pairs where the spacers separating the two motifs exhibit a high degree of sequence similarity; such motif pairs likely arise from duplications of the whole segment including the motifs and the spacer rather than due to selective constraints indicative of a functional importance of the motif pair. The method was used to scan 569 complete prokaryotic genomes for novel sequence motifs. Some motifs detected were previously known but other motifs found in the search appear to be novel. Selected motif pairs were subjected to further investigation and in some cases their possible biological functions were proposed.

**Conclusions:**

We present a new motif-finding technique that is applicable to scanning complete genomes for sequence motifs. The results from analysis of 569 genomes suggest that the method detects previously known motifs that are expected to be found as well as new motifs that are unlikely to be discovered by traditional motif-finding methods. We conclude that our approach to detection of significant motif pairs can complement existing motif-finding techniques in discovery of novel functional sequence motifs in complete genomes.

**Electronic supplementary material:**

The online version of this article (doi:10.1186/s12864-016-3400-0) contains supplementary material, which is available to authorized users.

## Background

Genomic DNA sequences contain numerous repetitive motifs or nucleotide sequence patterns that play various roles in the physiology of the cell, including regulation of gene expression, maintenance of DNA and its structure in the cell, recombination and other forms of mutation, and even recognition of DNA fragments for uptake from the extracellular space [1–5]. Repetitive motifs in DNA sequences can also encode functional elements in proteins and RNA. Consequently, a large amount of effort has been devoted to development of computational methods for detection of sequence motifs in DNA and protein sequences.

There are two major types of motif finding algorithms, namely supervised and unsupervised motif finding algorithms. The former methods require a sample of known occurrences of the motif and utilize this information in the search for additional motif occurrences in the analyzed sequence or sequences. The unsupervised methods, sometimes referred to as *ab initio* approach, do not require any prior knowledge about the motif sequences and detect novel sequence motifs that satisfy specified criteria (generally including unexpectedly high frequency of occurrence and high sequence similarity among different copies of the motif). In this article, we focus on unsupervised motif search. The unsupervised motif finding algorithms can be further classified into two major groups: 1) word-based methods that mostly rely on exhaustive enumeration, i.e., counting and comparing oligonucleotide frequencies and 2) probabilistic sequence models where the model parameters are estimated from sequences. Extensive work has been done on transcription factor binding site (TFBS) prediction during the past decades, driven by the obvious importance of these regulatory motifs in the organism’s physiology. Based on the type of DNA sequence information used by the TFBS finding algorithm, the methods could be classified into three major classes: 1) methods that use promoter sequences from coregulated genes from a single genome [6, 7], 2) methods that use orthologous promoter sequences of a single gene from multiple species [8–10] and 3) methods combining 1) and 2) [11, 12]. As a unified portal for online discovery and analysis of sequence motifs, the MEME Suite web server provides various tools in finding motifs representing features such as DNA binding sites and protein interaction domains [13].

While TFBS receive most attention among sequence motifs the repetitive sequence motifs in genomic DNA can have many other functions. We aim to expand the range of types of sequence motifs detected by motif-finding methods by searching for spaced motifs in complete genomes, which could arise, among other mechanism, from repetitive patterns in chromosome structure. The concept of searching for spaced sequence motifs is not new but the previous such methods generally aimed to detect TFBS, where spacing of the conserved segments of the motif is determined by the geometry of the DNA-protein interaction and generally does not exceed 6 or 7 bp (for example, refs [14, 15]). A more general approach implemented in HeliCis [16] allows detection of co-localized periodically spaced motifs, such as binding sites for multiple transcription factors. However, these methods are specifically designed for detection of TFBS in a collection of regulatory regions and are not suitable for scanning complete genomes. In contrast, our methodology is aimed at detection of DNA sequence motifs that can have diverse physiological functions, including regulatory motifs, motifs involved in DNA, RNA, and protein interactions, and motifs related to structural organization of DNA and/or the encoded proteins. Our approach is different from previous methods in that it searches for pairs of motifs that occur at a particular distance from each other significantly more often than expected without requiring that either of the motifs on its own occurs more frequently than expected. The goal is to detect paired motifs where the distance between the two motifs is under selective constraint but the specific sequence of the spacer is variable.

## Methods

### The motif-finding algorithm

The goal of the algorithm is to find motif pairs that have a preferred distance of separation. That is, they occur at a specific distance apart more often than expected by chance. The algorithm consists of the following steps:

1. Scan the genome and count the number of times that the pairs of motifs A and B (pentamers in the current application) occur at every spacer distance between 5 and 89 (inclusive). Record the total number as **n**;
2. Identify the distance (**d**) at which the pair has the maximum number of occurrences, record the maximum number as **fmax**;
3. Test whether the maximum number of occurrences **fmax** is large enough to claim significance of overrepresentation by surpassing some appropriate cutoff value **fcut**;
4. Repeat steps 1) – 3) for all 4^10^ possible pentamer pairs; record all significant pairs with fmax > fcut;
5. For each significant pair, align the spacers (the sequences separating the motifs A and B) pair-wise. Reduce **fmax** by 1 for each pair of aligned spacers with nucleotide identity greater than 70% and remove one of the two spacers from further comparisons with other spacers;
6. Identify and record motif pairs whose **fmax** is still above **fcut**. Record if the inverse complement is also significant with the same distance;
7. For motif pairs that satisfy the criteria above, investigate their distribution in the genome using Pattern Locator [17].

Step 1) consists of recording the total number of times a pair of pentamers A and B is found at the exact mutual distance **d**. The distance **d** is counted from the beginning of A to the beginning of B. Pairs with exactly the same spacers are counted only once (pairs with similar but not identical spacers are discounted later in step 5). The motivation for discounting motif pairs with identical or very similar spacers is that they likely arise from sequence duplication of the whole segment containing the motif pair and the spacer, which is not our primary goal; we aim to detect motif pairs where the distance between the motifs arises from selective constraints rather than as a result of sequence duplication events. The main reason we limit the motif spacer distance to 5–89 bp is due to growing computational complexity when ranges of paramters investigated are too large. Similar reasons apply to limiting the investigated motifs to pentamers; the number of possible pairs of hexamers is around 16 million (compared to ~1 million of possible pairs of all pentamers), which is prohibitive when the methodology is to be applied to hundreds of complete genomes as in this study. Another important motivation to use pentamers is related to how prokaryotic DNA-binding proteins, particularly transcription factors, interact with DNA. Many prokaryotic transcription factors interact with DNA in a sequence-specific manner via two alpha-helices that fit into adjacent turns of the major groove in the DNA double helix [18]. Consequently, although the whole binding site is often ~20 nucleotides long, it usually consists of two conserved segments of ~5 nucleotides where the protein directly interacts with the DNA bases, separated by a variable gap [19, 20].

After finding the most abundant distance **d** for each motif pair in step 2), statistical significance is assessed in step 3). For a given pair of motifs, if no specific distance is preferred, one would expect a uniform distribution of spacer distances within the whole range 5 to 89 bp. The problem of numbers of motif pairs found at different distances is analogous to the classical balls-in-urns problem where motif pairs represent the balls and different distances (spacer sizes) represent the urns. Suppose n balls are allocated to K urns at random, each ball being equally likely to fall into each urn, independently of any other balls. Let fmax denote the largest number of balls in any urn. This situation can be modeled with the uniform multinomial distribution in which there are n trials, each with K possible outcomes of equal probability.

David and Barton [21] showed that P(fmax < x) can be computed as

1 $$ \mathrm{P}\left(\mathrm{fmax}<\mathrm{x}\right)=\frac{n!}{K^n}\times \mathrm{The}\kern0.26em \mathrm{coefficient}\kern0.26em \mathrm{of}\kern0.26em {\uplambda}^{\mathrm{n}}\kern0.26em \mathrm{in}\kern0.26em {\left({\displaystyle {\sum}_{j=0}^{x-1}\frac{\uplambda j}{j!}}\right)}^{\mathrm{K}} $$

Subsequently, the *p-*value p = P(fmax > = x) = 1 - P(fmax < x).

The analogy in DNA sequences is straightforward with n being the total number of motif pair occurrences with spacer distance between 5 and 89 bp, K being the number of different distances (89–5 + 1 = 85) and fmax being the maximum number of occurrences of a specific distance within the range. A Bonferroni correction was applied to the *p-*value cutoff in order to account for multiple hypothesis testing (the test is performed for 4^10^, or ~1 million, different pairs of pentamers):

*p-*value-cutoff = 0.01 / number of motif pairs = 0.01 / 4^10^ ≈ 10^−8^.

Testing over multiple distances is accounted for in the uniform multinomial. SymPy, a Python library for symbolic mathematics, was utilized to perform the power expansion in formula (1) and to extract the coefficient needed to compute P(fmax < x). However, using SymPy to obtain the exact tail probability is computationally expensive, especially for large parameters of n and K. Hence, we used an approximation [22] to calculate the 100(1-α)% percentile of fmax with the following equation

2 $$ {\mathrm{F}}_{\upalpha}\approx \frac{n}{K}+\sqrt{\frac{n\; \log\;K}{K}}-\left( \log\; \log {\scriptscriptstyle \frac{1}{1-\alpha }}+1.266\right)\sqrt{\frac{n}{2K\; \log\;K}} $$

**Fig. 1:**
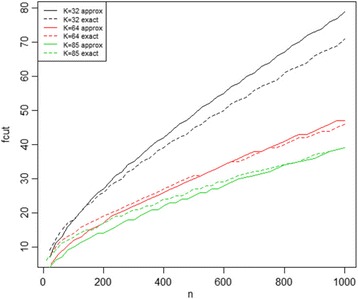
Comparison between approximate and exact *p-*value cutoffs.n is the total number of occurrences of some motif pair; fcut indicates the cutoff value corresponds to the selected p value threshold. Solid and dashed lines represent approximate values calculated by formula (2) and exact values computed with SymPy, respectively

In the formula above, F_α_ is the 100(1-α)% percentile of fmax; α is the desired Type I error rate; n is the total number of occurrences of the motif pair at hand with distance between 5 and 89, and K is the total number of distances investigated (K = 85). Note that the above approximation is based on an asymptotic theorem under certain regularities [23]. To verify that the approximation is sufficiently accurate with our set of parameters, comparisons between approximate and exact fcut values for different values of K were made using a fixed *p-*value 10^−8^ (Fig. 1). When K is relatively large (greater than 50), the equation (2) approximates the true values with reasonable accuracy as n gets large; however, when K is relatively small (smaller than 50), the equation tends to overestimate the true fcut values. Since the K used in this paper is 85, we decided to use exact cutoff values for n < = 600 while the approximated values are used for n > 600.

In Step 4], the steps 1 to 3 are repeated for all possible pentamer pairs. After all pentamer pairs are tested, the most promising candidates are selected for spacer alignment. In Step 1], only identical spacers could be recognized and eliminated but our goal is to discount all motif pairs which likely arose from recent duplication of the whole segment including the motif pair and the spacer. Consequently, sequence alignments are performed with NW-align, which is a simple and robust alignment program based on the standard Needleman-Wunsch algorithm [24], to eliminate potential duplicated spacers. A simple ‘greedy’ algorithm is applied to eliminate duplicated spacers. The first pair of spacers is aligned. If the nucleotide identity is ≥70%, then fmax is reduced by one and one of the spacers is excluded from further alignments. Additional pairs of spacers are subsequently aligned and excluded if the nucleotide identity exceeds 70%. This is continued until no spacers are more similar than the 70% cutoff. The value 70% was chosen as the lowest cutoff that gives a high confidence the sequence similarity is not coincidental; for example, the probability that two random sequences of ten nucleotides would match at seven positions (70% identity) is less than 0.01 and declines with increasing sequence length. We also performed tests with 65% and 75% cutoffs, which suggested that the cutoff 65% was too conservative whereas majority pentamer pairs identified with the 75% cutoff overlapped motifs detected also with the 70% cutoff (see Additional file 1: Table S1 for details). After this step, a motif pair whose fmax is still above fcut and whose inverse complement is also significant with the same distance is selected for further analysis. The reason for requiring a motif pair’s inverse complement also to be significant is that dispersed sequence motifs, which are the primary target of our search, are expected to be distributed in both DNA strands; consequently, both versions of the motif pair—direct and reverse—are expected to be over-represented in the genome. The subsequent analysis of the significant motif pairs is performed with Pattern Locator [25], which provides information on distribution of the motif pairs with respect to adjacent genes. Such information can be helpful in generating hypotheses about the motif pair’s possible function. Pattern Locator also reports the percentage of copies of the motif pair found in genes and intergenic regions (Additional file 2: Table S2), which allows easy identification of motifs that are most likely to have regulatory functions (predominantly intergenic motifs) and motifs that could reflect conserved patterns in amino acid sequences of the encoded proteins.

### DNA sequences

Annotated nucleotide sequences of complete prokaryotic genomes were downloaded from the NCBI FTP server [26]. We randomly selected only one genome per species when multiple strains of the same species were available. The final dataset included 569 genomes (Additional file 1: Table S1). For genomes consisting of multiple chromosomes the analysis was performed on all chromosomes.

## Results

The 569 prokaryotic genomes, each from a separate species, were scanned with the algorithm described above. In the 569 genomes, 3326 motif pairs were identified as significant by our criteria (Additional file 2: Table S2). The summary statistics for selected characteristics of the significant motif pairs are shown in Table 1.

**Table 1:** Summary statistics of 3326 motif pairs identified in 569 genomes

	Min	1st Qu.	Median	Mean	3rd Qu.	Max
d	5	19	43	39.9	53	89
Initial_n	6	41	62	80.6	100	644
Reduced_n	6	27	44	57.8	73	633
Difference	0	2	9	22.7	26	335
Cutoff	6	18	31	47.8	64	521
Reduced_n/Cutoff	1	1.06	1.16	1.33	1.42	4.93
Gene	0%	20%	81%	61%	96%	100%
Intergenic	0%	2%	12%	35%	73%	100%
Overlap	0%	0%	2%	4%	4%	100%

The summary statistics is per motif. Meaning of abbreviations in the table: d, spacer length of a motif pair; Initial_n, number of copies of the motif pair before alignment; Reduced_n, number of copies after alignment and elimination of duplicate spacers; Difference, the difference between Initial_n and Reduced_n; Cutoff, significance cutoff (the lowest number of copies for the motif pair to be considered significant); Reduced_n/Cutoff, the ratio of Reduced_n and Cutoff, indicating the relative significance for the motif pair; Gene, the percentage of each motif pair occurrences found in genes; Intergenic, the percentage of each motif pair occurrences that are in intergenic regions; Overlap, the percentage of each motif pair occurrences that overlap with a gene start or end. These percentages are calculated as follows: For any given significant motif, we run a query with Pattern Locator, which gives the percentage of the motif occurrences that fall in gene, intergenic region or overlap with gene starts or ends. The quantiles in the table are for these percentages over all significant motifs

**Fig. 2:**
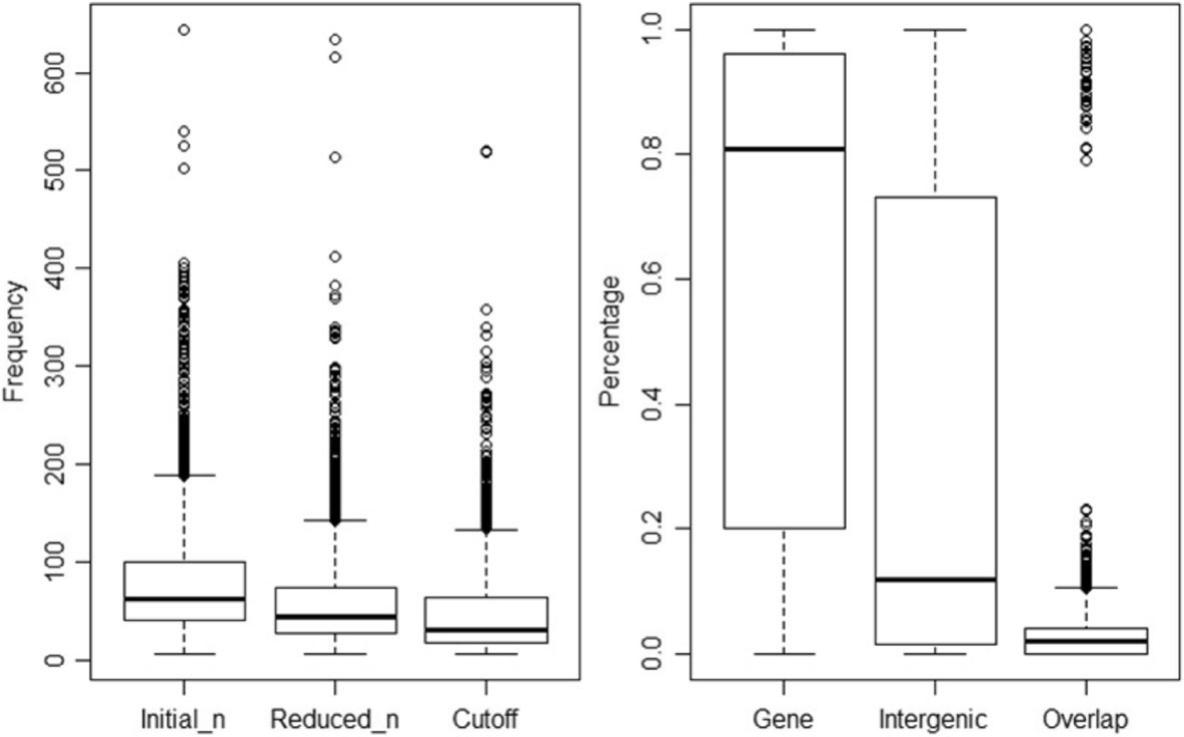
Distribution of key motif statistics. Frequency is number of copies of each significant motif pair; Percentage is the percentage of significant motif pairs found in corresponding region

**Fig. 3:**
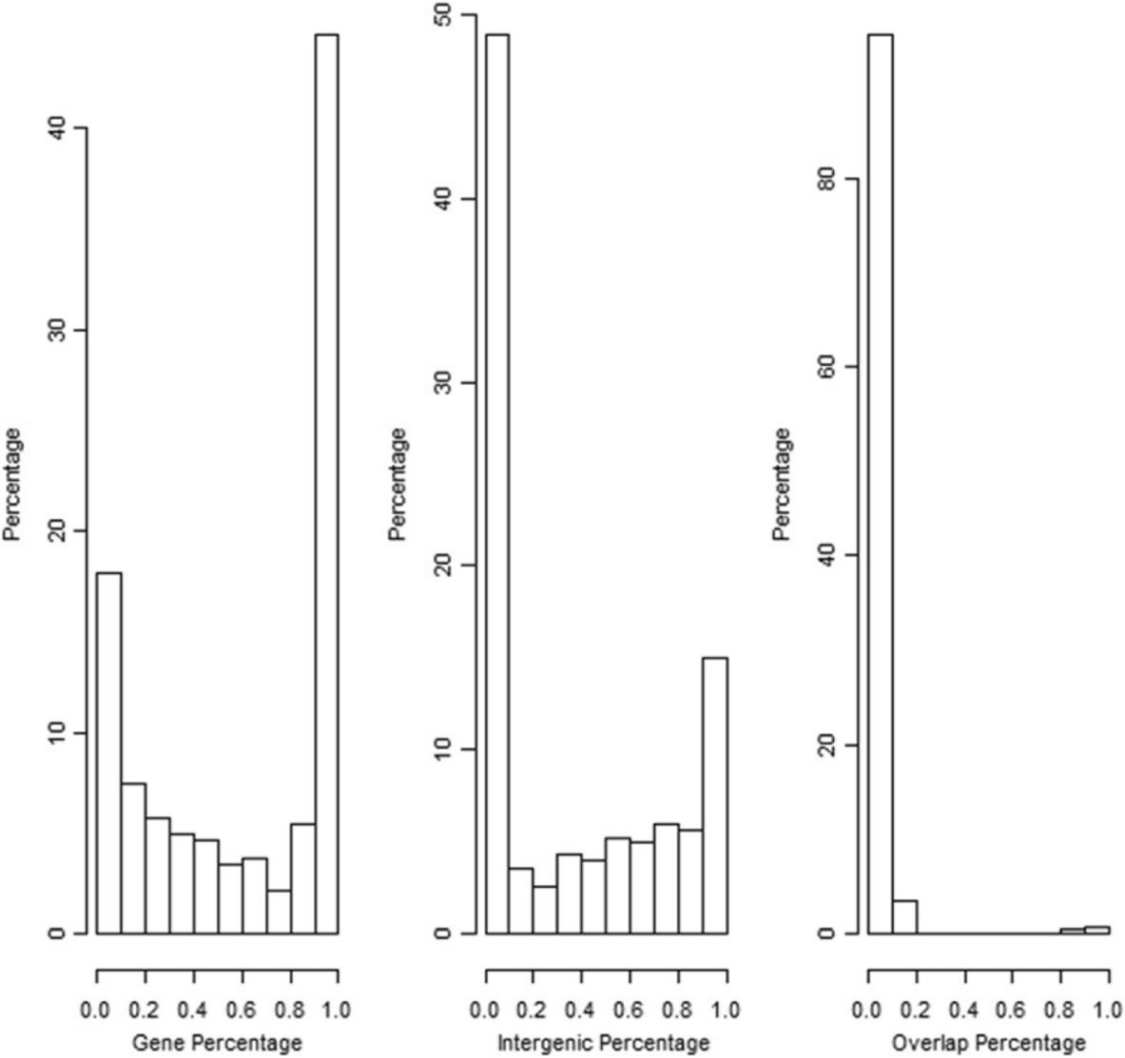
Distribution of motif pairs with respect to gene and inter-genic region. In each plot, the horizontal axis indicates the percentage of occurrences of significant pairs found in corresponding region

Distributions of some key statistics are further demonstrated in Figs. 2 and 3. The right panel in Fig. 2 indicates that median percentage of significant pairs found in protein- coding regions is around 80%, which is close to the fraction of protein-coding region in a typical prokaryotic genome; in other words, the significant motif pairs do not show a strong overall bias for protein-coding regions or intergenic regions. In Fig. 3, the first two plots show that the distributions of motif pairs are bi-modal in both gene and intergenic region. 50 percent of significant motif pairs occurred in genes more than 90% of the time and 25 percent occurred in genes less than 10% of the time, whereas fewer motif pairs are distributed approximately evenly among both genes and intergenic regions. In other words, majority of the significant motifs show a strong preference for either genes or intergenic regions. The same conclusion can be reached upon inspection of the raw data in Additional file 3: Table S3.

**Fig. 4:**
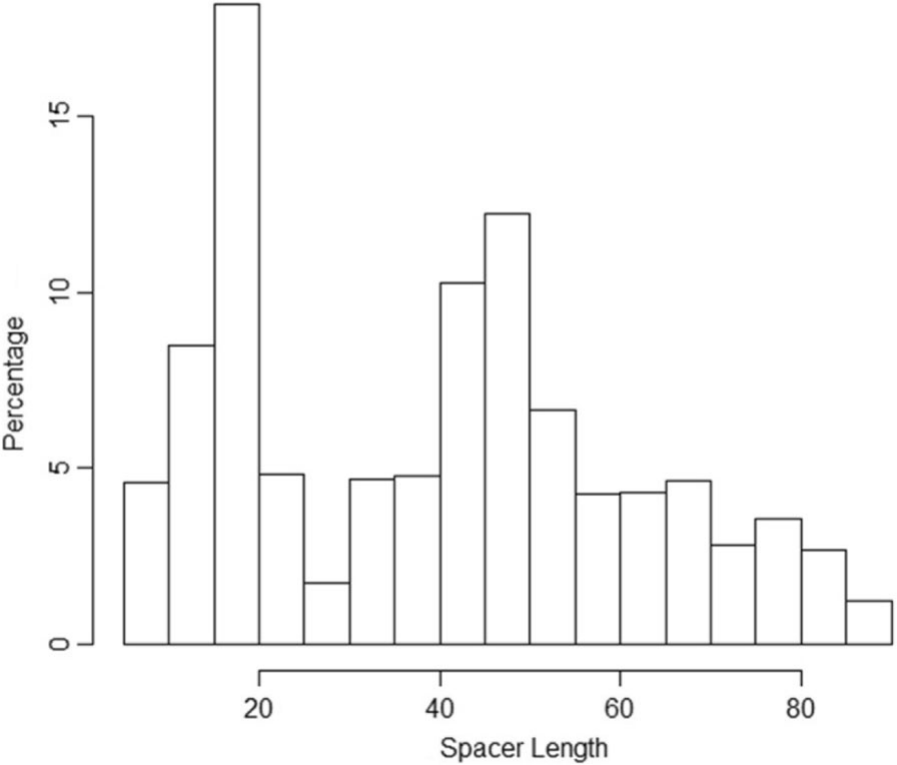
Spacer Length Distribution of Candidate Pairs

The distribution of the spacer length for the motif pairs is shown in Fig. 4. There are two peaks—one is around spacer lengths 15 to 20 bp and the other one is around 40 to 50 bp. The reason for the first peak is partly due to a conserved palindromic motif pair (CGAAA and TTTCG with a spacer length of 19 bp), which is widely distributed among a variety of genes of *Mycobacterium* species. In total, approximately 150 motif pairs conforming to this consensus were identified in the analyzed *Mycobacterium* genomes out of 701 motif pairs with spacer length 15 to 20 bp found in all analyzed genomes. The peak at 40–50 bp reflects wide distribution of clustered regularly interspaced short palindromic repeats (CRISPRs) among the analyzed genomes. CRISPRs are arrays of ~30 bp perfect repeats separated by ~30–40 bp variable spacers, which give rise to many significantly overrepresented motif pairs with this spacer size [27] (see also below).

Another noteworthy statistic in Table 1 is the difference between the initial number of copies of a significant motif pair (Initial_n) and the number of copies remaining after elimination of duplicate spacers (Reduced_n). The mean value of Initial_n is 80.6 whereas Reduced_n has mean 57.8, indicating that more than 25% of occurrences of all significant motif pairs in the genomes may have arisen from sequence duplication events. This confirms the importance of removing the “false positives” resulting from sequence duplications when searching for candidate motif pairs whose specific relative positioning is maintain by selective constraints.

All significant motif pairs found in the analyzed genomes are reported in Additional file 3: Table S3. In total, 307 genomes have at least one significant motif pair identified. Among those, *Rhodoferax ferrireducens* T118 has the highest number of pairs detected, 140 (Additional file 3: Table S3).

Thirty genomes (listed in Additional file 4: Table S4) were selected for in-depth investigation of the identified significant motif pairs. Some of the significant motif pairs were found to be related to previously known motifs such as CRISPR or Shine-Dalgarno sequence whereas others appear to be potentially novel, previously uncharacterized sequence motifs. Majority of the identified motif pairs can be classified in one of the five categories, namely CRISPR-related, Rho-independent transcription terminators, tRNA-related motifs, motifs containing the Shine-Dalgarno sequence, and protein-related motifs.

### CRISPR-related motif pairs

CRISPRs are segments of prokaryotic DNA containing periodic repetitions of ~30 nucleotides. Each repetition is followed by short segments of “spacer DNA”, typically 30–40 nucleotides long. The spacers are short fragments of nucleotide sequence from phages or plasmids to which the bacterium had been exposed in the past [28]. These fragments in conjunction with the Cas proteins are subsequently used to recognize and destroy the DNA of the same phages or plasmids in future encounters. The CRISPR/Cas system is a form of prokaryotic immune system that confers resistance to foreign genetic elements such as plasmids and phages by recognizing and digesting them [29]. CRISPRs are found in approximately 40% of sequenced bacterial genomes and 90% of sequenced archaea [30]. Given its properties of regularly interspaced repeats with spacers of variable sequence but constant length, it’s not surprising that parts of CRISPRs are among the paired motifs detected by the algorithm.

Table 2 lists significant motif pairs found in the 30 genomes subjected to in-depth analysis that are related to CRISPR. Results were verified by comparison with CRISPRdb, a comprehensive database of known CRISPR occurrences [30]. Candidatus *Desulforudis audaxviator* MP104C contains two types of CRISPR sequences, GTTTCAATCCCTCGTAGGTAGGCTGGAAAC and CTTTCAGTCCCCTTTTCGTCGGGTCGGTCGCTGAAAC. The first four patterns listed in Table 2 for this genome correspond to the former whereas the last three reflect the latter CRISPR sequence. The patterns consist of two pentamers near the ends of the CRISPR sequence and the spacers, which are variable in sequence bur have a constant length, are responsible for the fixed distance between the pentamers. For patterns detected in *Clostridium thermocellum* ATCC 27405, the first two arise from repeats GTTTCAATTCCTCATAGGTACGATAAAAAC and the rest are due to GTTT(G/T)TATCGTACCTATGAGGAATTGAAAC. All patterns found in *Corynebacterium aurimucosum* ATCC 700975 are due to the CRISPR sequence GTGCTCCCCGCGTAAGCGGGGATGAGCC. Similarly, all patterns detected in the other two *Corynebacterium* species are due to the same consensus GGCTCATCCCCGCTGGCGCGGGGAGCAC.

**Table 2:** Significant motif pairs related to CRISPR repeats

Genome	Pattern
Anaeromyxobacter dehalogenans 2CP 1	GGGGA(N)_43_TCCCC
Candidatus Desulforudis audaxviator MP104C	AAACG(N)_35_GTTTC GAAAC(N)_35_GGTTT AAACT(N)_35_GTTTC GAAAC(N)_36_GTTTC TGAAA(N)_39_TTTCA CTGAA(N)_40_TTTCA CTGAA(N)_41_TTCAG
Clostridium thermocellum ATCC 27405	TACGA(N)_52_CCTCA GTACG(N)_52_TCCTC ATGAG(N)_53_TCGTA TGAGG(N)_53_CGTAC ATGAG(N)_54_CGTAC ACCTA(N)_55_TATCG CCTAT(N)_56_TCGTA ACCTA(N)_56_ATCGT CTATG(N)_56_CGTAC
Corynebacterium aurimucosum ATCC 700975	GGGGA(N)_43_TCCCC CGGGG(N)_44_TCCCC CGGGG(N)_45_CCCCG GGGGA(N)_45_CCCGC CGGGG(N)_46_CCCGC
Corynebacterium jeikeium K411	GGGGA(N)_43_TCCCC CGGGG(N)_44_TCCCC CGGGG(N)_45_CCCCG GGGGA(N)_45_CCCGC CGGGG(N)_46_CCCGC
Corynebacterium urealyticum DSM 7109	GGGGA(N)_43_TCCCC CGGGG(N)_44_TCCCC GGGGA(N)_45_CCCGC CGGGG(N)_45_CCCCG

Pattern X_1_X_2_X_3_X_4_X_5_(N)_D_Y_1_Y_2_Y_3_Y_4_Y_5_ denotes 5-mer X_1_X_2_X_3_X_4_X_5_ is followed by 5-mer Y_1_Y_2_Y_3_Y_4_Y_5_ with D nucleotides apart; for non-palindromic patterns, only sequence in one DNA strand is listed

Note that our software is not intended for detection of CRISPR sequences and the detection of CRISPR-related sequence pairs are a byproduct of the characteristic CRISPR structure. Our method is designed for detection of dispersed sequence motifs, whereas CRISPRs consist of repeats clustered at a small number of loci in the genome. This explains why CRISPR-related motif pairs were not detected in many genomes with CRISPR. The main reason is the requirement that both direct and reverse complement versions of a motif pair independently satisfy the criteria for statistical significance (see Methods). Many genomes contain a single CRISPR locus, which means that all CRISPR sequences are in the same orientation with respect to the direct and reverse strands. Such CRIPSR loci can only be detected if they contain a palindromic pair of pentamers that satisfies all other criteria.

### Rho-independent transcription terminators

A transcription terminator is a functional sequence located at the 3′ end of genes that mediates the transcriptional termination. In prokaryotes, there are two types of terminators, namely Rho-dependent and Rho-independent terminators. The former require Rho factor to terminate the transcription process while the latter one forms a self-annealing hairpin structure to serve the purpose. The terminator sequence generally contains a GC-rich region of dyad symmetry followed by a short poly-T tract [31].

Motif pairs related to transcription terminators were found in 2 out of the 30 genomes (Table 3). The results were verified by TransTermHP, which is a specialized software to find rho-independent transcription terminators in bacterial genomes [32]. In *Actinobacillus pleuropneumoniae* the pattern is a high-G + C palindrome; in *Haemophilus influenzae* the pattern detected by our method is not a palindrome, but instead includes a poly-T tract. Both features in the two patterns identified correspond to the standard structure of Rho-independent terminators, which consist of a GC-rich palindrome followed by a short poly-T tract.

**Table 3:** Motif pairs that are part of transcription terminators

Genome	Pattern
Actinobacillus pleuropneumoniae serovar 5b L20	CAAAA(N)_13_TGACC CAAAA(N)_15_ACCGC GGTCT(N)_15_TTGCA TGCAA(N)_15_CGACC TGCAA(N)_16_AACCG GCAAA(N)_16_ACCGC TTGCA(N)_16_AGACC GCGGT(N)_16_TTTAC GCAAA(N)_17_CCGCT CGGTC(N)_17_TGCAA TTTGC(N)_18_GACCG AAGCG(N)_19_TTGCA GCGGT(N)_19_GCAAA TTTTG(N)_19_GACCG CGGTC(N)_38_GACCG AGCGG(N)_40_GACCG
Haemophilus influenzae Rd KW20	GGTCA(N)_10_TGTTT GGTCA(N)_11_GTTTT CGGTC(N)_12_GTTTT GCGGT(N)_12_AGTTT GCGGT(N)_12_TGTTT GGTCA(N)_12_TTTTT CGGTT(N)_12_GTTTT TGCGG(N)_13_AGTTT CGGTC(N)_13_TTTTT GCGGT(N)_13_GTTTT TGCGG(N)_13_TGTTT CGGTA(N)_13_TTTTT CGGTT(N)_13_TTTTT GCGGT(N)_13_ATTTT GCGGT(N)_14_TTTTT GTGCG(N)_14_TGTTT TGCGG(N)_14_GTTTT TGCGG(N)_15_TTTTT AGTGC(N)_15_TGTTT GTGCG(N)_15_GTTTT GCGGT(N)_15_TTTTG AGTGC(N)_16_GTTTT AAACA(N)_16_CACTT TGCGG(N)_16_TTTTG GTGCG(N)_16_TTTTT

The fact that transcription terminator-related motif pairs were detected by our program is actually unexpected because the terminators are characterized by the stem-loop secondary structure but not a specific repeated sequence. This is also the reason why we did not detect transcription terminator-related motif pairs in other genomes. However, the *H. influenzae* genome contains about 1300 copies of the uptake signal sequence with the consensus AAGTGCGGT and terminators in *H. influenzae* are often composed of two copies of the USS in the opposite orientations that form the stem of the stem-loop structure [3]. That leads to prevalence of this specific sequence in the terminator stem-loop structures and it is the reason why we are detecting terminators in *H. influenzae*. Analysis of the *A. pleuropneumoniae* genome with the AIMIE software [33] revealed an abundant motif AAGCGGT, which is similar to the USS in *H. influenzae* and may have an analogous function [34]. Like the USS in *H. influenzae*, the *A. pleuropneumoniae* motif frequently occurs in dyad pairs found at the 3′ ends of genes, which probably serve as transcription terminators.

### tRNA-related motifs

tRNA-related motifs were detected in four genomes – 3 of them are inside tRNA genes and correspond to parts of a conserved pattern TAGAGC(N)_27_GGTTCG near the 5′ end of the gene (Table 4). Parts of this pattern are highly conserved in tRNA genes from diverse species [35]. However, the motif pairs in *A. butzleri*, *S. muelleri*, and *N. profundicola* just exceed the significance cutoff of our method and they are not detected in other genomes because the number of copies is not statistically significant.

**Table 4:** tRNA-related motifs identified

Genome	Pattern	tRNA related
*Arcobacter butzleri* RM4018	AGAGC(N)_27_GTTCG	Inside tRNA
Candidatus *Sulcia muelleri* GWSS	TAGAG(N)_27_GGTTC	Inside tRNA
*Nautilia profundicola* AmH	TAGAG(N)_28_GTTCG	Inside tRNA
*Methanopyrus kandleri* AV19	CGTTA(N)_9_TAACG	tRNA downstream

**Fig. 5:**
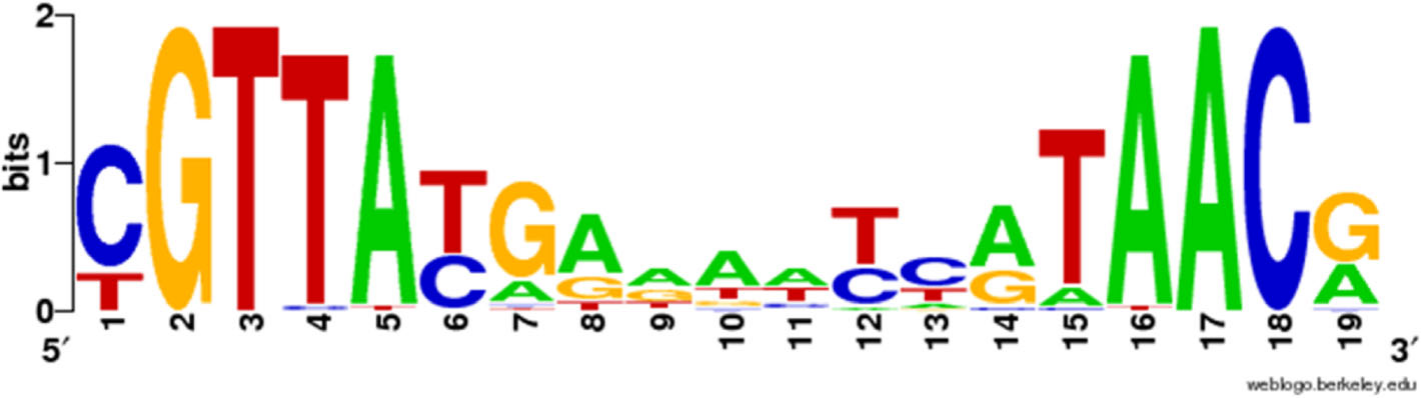
Sequence logo for motif pair downstream of tRNA genes in *M. kandleri*

The pattern in *Methanopyrus kandleri* AV19 differs from the other three in that it resides outside of tRNA genes, specifically about 10 base pairs downstream of the tRNA genes. Other tRNA genes (about half of the total number of tRNA genes in this genome) have similar patterns at the same position (10 bp from the end of tRNA gene). The consensus sequence of the motif is shown in Fig. 5.

The sequence is palindromic with positions 2–5 and 15–18 being the most conserved. Unlike the other tRNA-related motif pairs, this motif appears to be specific to *M. kandleri* and we did not find similar motifs in other genomes. The methanogenic archaeon *M. kandleri* grows at temperatures of 84–110 °C and genome analyses revealed strategies to adapt to these harsh conditions. Among other adaptations, *M. kandleri* uses a unique tRNA C-to-U editing mechanism at base 8 for 30 different tRNA species [36]. We speculate that the palindrome discovered by our software might serve as a target signal for the 3′ maturation enzyme and its presence specifically in *M. kandleri* may be related to the unique mechanism of tRNA maturation employed by this organism.

### Motifs related to Shine-Dalgarno sequence

Located around 8 bp upstream of the start codon, Shine-Dalgarno (SD) sequence serves as ribosome binding site in mRNA of prokaryotic genomes [37]. With a 6-base consensus sequence of **AGGAGG**, the SD sequence recruits and aligns ribosome to mRNA in order to initiate translation [38].

Motif pairs related to SD sequences were found in seven of the 30 investigated genomes. The left part of the motif pair corresponds to the SD sequence whereas the right part contains the start codon. The SD-related motif pairs arise from the SD sequence being located at the same distance from the start codon in many genes (Table 5). The two *Campylobacter* species and *Helicobacter hepaticus* have exactly the same patterns, as do the two *Thermosipho* species. *Exiguobacterium sibiricum* only differs from the *Thermosipho* at one position ahead of start codon (C instead of T); actually all 7 patterns are similar. Interestingly, the start codon ATG in all these patterns is followed by A or AA, suggesting that this start codon context is preferred in these genomes. We speculate that the primary reasons why SD-related motif pairs are found in only seven of the 30 investigated genomes is that the percentage of genes with recognizable SD sequences varies widely among different species [39] and the start codon context is also variable, which leads to significant overrepresentation of exact pentamer pairs in some but not all genomes.

**Table 5:** Genomes with Shine-Dalgarno sequence identified

Genome	Pattern
Campylobacter concisus 13826	AAGGA(N)_6_ **ATG**AA
Campylobacter curvus 525 92	AAGGA(N)_6_ **ATG**AA
Exiguobacterium sibiricum 255 15	GGAGG(N)_6_C**ATG**A
Helicobacter hepaticus ATCC 51449	AAGGA(N)_6_ **ATG**AA
Lactobacillus plantarum JDM1	AGGAG(N)_9_ **ATG**AA
Thermosipho africanus TCF52B	GGAGG(N)_6_T**ATG**A
Thermosipho melanesiensis BI429	GGAGG(N)_6_T**ATG**A

Start codon is shown in bold

### Protein-related motifs

The last major class of motif pairs detected are protein-related motifs, which reside mostly in protein-coding genes. The list of such motifs found in the 30 genomes that were investigated in detail is provided in Table 6. Two types of proteins stand out: one is ABC transporter ATP-binding protein and the other one is PE/PPE family protein.

**Table 6:** Genomes with significant protein-related motif pairs

Genome	Pattern	Protein related
*Acidovorax citrulli* AAC00 1	**TAC**GA(N) _59_AC**GAC**	Inside YD repeat-containing protein
*Anaeromyxobacter dehalogenans* 2CP 1	AC**TGC**(N)_6_ **TGC**CA	Inside cytochrome c
*Geobacter bemidjiensis* Bem	A(G/C)**TGC**(N)_6_ **TGC**CA	Inside cytochrome c
*Clostridium beijerinckii* NCIMB 8052	T**GGT**A(N)_55_T**GGT**A	Inside cell wall binding repeat-containing protein
*Leuconostoc mesenteroides* ATCC 8293	**GGT**GG(N)_64_ **GAA**CC	Inside ABC transporter ATPase
*Staphylococcus aureus* RF122	**GGT**GG(N)_61_ **GAT**GA **GGT**GG(N)_64_ **GAA**CC	Inside ATP-binding ABC transporter protein
*Streptococcus dysgalactiae* equisimilis GGS 124	**GGT**GG(N)_61_ **GAT**GA	Inside ABC transporter ATP-binding protein
*Streptococcus equi* 4047	**GGT**GG(N)_61_ **GAT**GA	Inside ABC transporter ATP-binding protein
*Mycobacterium bovis* AF2122 97	G**ATC**A(N)_33_ TG**ATC**	Inside PE-PGRS family protein
*Mycobacterium marinum* M	**TAT**CA(N)_70_ **TAT**GC	Inside PE-PGRS family protein
*Mycobacterium marinum* M	**AAC**TC(N)_25_ **AAC**TC	Inside PPE family protein
*Mycobacterium tuberculosis* CDC1551	G**ATC**A(N)_33_ TG**ATC**	Inside PE-PGRS family protein
*Ralstonia eutropha* H16	**CAC**CT(N)_52_ **TAC**AA	Inside extra-cytoplasmic solute receptor

Complete codons are highlighted in bold face

ABC transporters are transmembrane proteins that utilize ATP hydrolysis for translocation of various substrates across membranes and are widespread in all phyla from prokaryotes to humans [40]..Motif pairs related to ABC transporter ATP-binding subunits were found in 4 different species. The motif pair GGTGG(N)_64_GAACC is significant in *Leuconostoc mesenteroides* and *Staphylococcus aureus* whereas two *Streptococcus* species have a significant motif pair GGTGG(N)_61_GATGA (Table 6). When combined, the motif pairs reveal an extended consensus GGTGG(A/T)(N)_60_GA(T/C)GAACC, which translates to an amino acid motif GG-x(20)-DEP, where G, D, E, and P denote glycine, aspartic acid, glutamic acid and proline, respectively, and x denotes any amino acid. The GG is part of the characteristic ABC transporter motif LSGGQ and the DEP is part of the Walker B box, which is highly conserved in the ABC transporter protein family [41].

The PE and PPE proteins were first reported in the genome sequence of *Mycobacterium tuberculosis* strain H37Rv and were subsequently identified in all mycobacterial species as well as some *Rhodococcus* and *Nocardia farcinica* genomes [42]. The proteins are characterized by presence of a PE or PPE domain, respectively, which is a 225 amino acid residue conserved region located near the C-terminus. The PE/PPE domain comprises a pentapeptide sequence motif GxSxG/S at the N-terminus and conserved amino acid residues Ser, Asp and His [42]. However, the conservation of the patterns found in *Mycobacterium* is due to conserved amino acids in the protein sequence other than this signature domain. The pattern in *A. citrulli* arises from Y(TAC)D(GAC) repeats in the protein. Motifs found in cytochrome c translate to CxxCH, which is highly conserved in this protein.

### Potential novel motifs and other motifs of interest

While many motif pairs detected by our algorithm can be traced to known motifs with known biological functions, the method also shows potential in detecting novel motifs, such as the motif pair located downstream of tRNA genes in *Methanopyrus kandleri* that might serve as target signal for tRNA gene 3′ end maturization enzyme. Other potential novel motifs include the motif pair CGAAA(N)_19_TTTCG in *Mycobacterium* species and some other motifs located in protein coding genes that are not due to known conserved amino acids motifs (Table 6). We selected a short list of promising potential novel motifs from Additional file 2: Table S2 based on the spacer distance and the percentage of copies in intergenic regions (Table 7). The rationale is that the motifs with regulatory functions are likely to reside in intergenic regions whereas motif pairs in protein coding regions are more likely to be related to properties of the encoded proteins, which, while not without interest, are not the main target of this work. The other criterion is that we chose motif pairs with spacer lengths different from known repeats, such as the CRIPSPR-related motifs. Properties of each of these motif pairs based on the analysis with Pattern Locator (http://www.cmbl.uga.edu/software/patloc.html) are briefly summarized in Table 7.

**Table 7:** Selected potential novel motifs

Genome	Motif Pair^a^	Gene	Intergenic	Overlap
*Agrobacterium radiobacter* K84	TTAAT(N)_5_ATTAA	0%	100%	0%
*Bifidobacterium animalis* lactis AD011	GGGACAG(N)_15_TGTCCC	0%	95%	5%
*Desulfatibacillum alkenivorans* AK 01	CCTAC(N)_19_TAGGT	3%	97%	0%
*Clostridium kluyveri* DSM 555	TTGAC(N)_19_ATAAT	16%	83%	1%
*Acidovorax ebreus* TPSY	GCTTAT(N)_5_AAGCG	4%	94%	2%
*Bradyrhizobium japonicum* USDA 110	GAATCCAT(N)_23_ATGGATTC	0%	90%	10%
*Polaromonas naphthalenivorans* CJ2	ATAGCT(N)_22_CAAAAG	14%	81%	5%
*Petrotoga mobilis* SJ95	ATTATA(N)_18_GTCAA	5%	86%	9%

^a^ Significant motif pairs comprised of overlapping pentamers were combined and reported as a single motif pair

The motif pair TTAAT(N)_5_ATTAA of *A. Radiobacter* has 9 copies in the genome, all of which are located in intergenic regions and mostly about 100 bp upstream of 5′ ends of genes (translation start sites). That indicates their potential functions in gene regulation. Queries to PRODORIC [43], a database of prokaryotic regulatory interactions, did not produce a match to a motif of the same or similar consensus sequence.

The motif pair CCTAC(N)_19_TAGGT in *D. alkenivorans* has 57 copies with 55 of them located in intergenic regions. The distances to nearest gene vary substantially from less than 50 bp to over 500 bp. Eighteen copies of the motif are located between convergent genes (that is, the motif is downstream of the 3′ end with respect to both adjacent genes), which argues against a possible role in regulation of transcription initiation. There is no obvious relationship to genes related to any particular function.

The GCTTAT(N)_5_AAGCG motif pair in *A. ebreus* has 114 copies in the genome of which 5 are in genes, 107 are in intergenic regions and 2 partially overlap with genes. Orientation of adjacent genes for intergenic patterns are 44 convergent (−> <−), 4 divergent (<− −>) and 59 co-oriented(<− <− or −> −>). Whether the patterns are located upstream or downstream, their distances to the 5′ or 3′ ends of genes are generally small (less than 50 bp). In an analogy to the motif pair in *D. alkenivorans*, frequent occurrence between convergently transcribed genes argues against a possible role in transcription initiation.

The motif GAATCCAT(N)_23_ATGGATTC in *B. japonicum* has 52 copies with 47 of them in intergenic regions and 5 overlapping with 5′ ends of genes. Almost all motif pairs are proximal (~50 bp) to the 3′ end of a gene, indicating that they might be involved in transcription termination, although the gap between the two parts of this palindromic motif (23 bp) is larger than the typical loop size in the hairpin structures formed by Rho-independent transcription terminators. Majority of the adjacent genes encode hypothetical proteins of unknown function.

The motif pair ATAGCT(N)_22_CAAAAG in P. *naphthalenivorans* has 125 copies in the genome of which 101 are in intergenic regions, 18 in genes and 6 overlap with a gene’s start or end. 34 copies are between convergently transcribed genes, again suggesting that the main role of this motif pair is not related to transcription initiation. Similar to the GCTTAT(N)_5_AAGCG motif pair in *A. ebreus*, the 101 copies in intergenic regions are often close (~50 bp) to adjacent genes.

The motif pair GGGACAG(N)_15_TGTCCC of *B. animalis* has 21 copies in the genome, one of which overlaps with a 5′ end of a gene and the other 20 are in intergenic regions. Ten copies of the motif pair feature at least one adjacent gene involved in amino acid metabolism or amino acid modifications suggesting a potential role in amino acid metabolism. However, most copies of the motif are not proximal to 5′ ends of genes (and mostly closer to a 3′ end of a gene) probably arguing against a role in transcription initiation.

The ATTATA(N)_18_GTCAA motif pair of *P. mobilis* (inverted complement TTGAC(N)_18_TATAAT) has 56 copies in the genome of which 48 are in intergenic regions, three are in genes, one partially overlaps with the 5′ end of a gene, and four overlap with the 3′ end of a gene. Notably, 12 copies are within 50 bp upstream of a tRNA gene. Four additional copies are next to ribosomal protein genes and 12 are next to genes annotated as transcription regulators. The motif conforms to the consensus −35 and −10 sites of bacterial promoters and the precise adherence of these specific genes to the promoter consensus probably reflects the importance of these genes and their efficient transcription. The motif pair TTGAC(N)_19_ATAAT in *C. kluyveri* also matches the consensus −35 and −10 site sequences. It has 94 copies of which 78 are in intergenic regions, 15 are in genes 1 is overlap with gene. Over 1/3 of the copies found in intergenic regions are within 50 bp from the 5′ end of genes.

## Discussion

*Ab initio* detection of candidate functional sequence motifs in complete genomes traditionally relies on word-counting approaches, which follow from a reasoning that selective constraints on a functional sequence motif could lead to statistically significant excess (or deficit) of the motif occurrences in the genome. Consequently, such methods aim to detect specific words (oligonucleotides, substrings…) in DNA sequence that occur significantly more or less often than expected in some null model, generally based on a Markov chain representation of the sequence [33, 44–46]. Other commonly used unsupervised motif-finding methods utilize a probabilistic model of the motif, which allows formulating the motif-finding problem as an optimization task. The optimization is typically solved by one of the Markov chain Monte Carlo (MCMC) class of methods [47–49]. However, the latter methods are generally applied to a collection of relatively short sequences (such as putative promoters) and are not well suited for application to complete genomes.

Our approach is similar to word-counting methods but differs from methods described above in aiming to detect pairs of motifs that exhibit a significant preference for occurrence at a specific distance from each other rather than unexpectedly high occurrence relative to some null model of a random sequence. Another important feature of our algorithm is exclusion of motif pairs that feature identical or similar spacers. This step is designed to filter out motif pairs that arise from duplications of sequence fragments containing the motif pair as well as the spacer sequence, such as those resulting from proliferation of transposons, duplicated genes, or other forms of (large) sequence repeats. Consequently, our method specifically targets motif pairs that are likely maintained by selective constraints acting on the specific relative distance of the motifs, unlike motifs that arise from sequence duplication and may not be functional *per se*.

The current implementation of the method that was tested in this work is limited to pairs of pentanucleotides and spacer lengths up to 85 nucleotides. These limitations are partially driven by considerations of computational complexity of applying the software for analysis of hundreds of complete genomes. However, the pentanucleotides are also a reasonable choice with respect to DNA-protein interactions, where the sequence-specific bonds form between the exposed amino acid side chains and the base pairs in the DNA major groove, often leading to patterns of four to five adjacent conserved positions [50]. Moreover, pairs of motifs longer than five nucleotides can still be detected as pairs of overlapping pentanucleotides (Tables 2, 3, and 7).

Investigating locations of the detected motif pairs relative to genes and other known functional elements can often provide hints regarding their possible physiological roles or at the minimum help exclude some possible functions. For that purpose, our motif detection pipeline is combined with Pattern Locator, which provides basic statistics on locations of sequence patterns in the genome [17]. Detailed investigation of motif pairs found in 30 selected genomes allowed us to link some of the motif pairs to known biological functions, such as Rho-independent transcription terminators, CRISPR elements, and combinations of the transcription factor binding site and translation initiation site (start codon), which occur at specific distance from each other (Tables 2, 3, and 5). A number of conserved motif pairs were found in genes and we speculate that many of such patterns can arise from constraints on the properties of the encoded proteins, although only some of these patterns are related to previously known protein sequence motifs (Table 6).

A novel motif pair detected in *Methanopyrus kandleri* is of particular interest due to its location downstream of tRNA genes (Fig. 5). This motif is unlikely to be detected by motif-finding software designed for detection of transcription factor binding sites because this particular motif is located outside the regulatory regions where transcription factor sites generally reside. The word-counting methods that are more suitable for scanning complete genomes for overrepresented sequence motifs are also unlikely to detect such a motif due to their low sensitivity to spaced motif pairs separated by a variable spacer. Due to the unique mechanism of tRNA maturation in *M. kandleri* [36], we speculate that this motif could be involved in the tRNA maturation process, possibly as a binding site for the maturation enzyme.

It is important to note that the motif pairs listed in Tables 2, 3, 4, 5 and 6 represent only a fraction of the significant motif pairs detected by our methodology, namely those that we were able to link to a likely physiological function. The full set of motif pairs detected in 569 genomes (Additional file 2: Table S2) contains novel motifs that may not be detectable by other methods. Due to the conservative criteria for the detection of motif pairs and filtering of motif pairs with conserved spacers, we proffer that many of the motif pairs listed in Additional file 2: Table S2 arise from selective constraints on the relative positions of the motifs. Such motif pairs could play important physiological roles and at the same time escape detection by standard motif-finding techniques. A subset of selected candidate novel motifs (Table 7) was subjected to additional analysis that allows narrowing down a possible range of functions of these motifs. We conclude that method for detection of significant motif pairs described above is capable of detection of novel functional elements that are unlikely to be discovered by other method and we offer the paired-motif methodology as a tool to complement mainstream motif-finding methods for detection of functional elements in complete genomes.

## Conclusions

We present a novel motif-finding method based on detection of pairs of sequence motifs with statistically significant preference for a specific distance from each other. The method is suitable for application to long DNA sequences including complete genomes. Application to 569 prokaryotic genomes yielded a list of 3326 significant motif pairs (pairs of pentamers in the present implementation of the method) including many that are related to sequence motifs of known functions as well as novel sequence motifs. The complete list of all significant motif pairs found in the 569 genomes is available in Additional file 2: Table S2. We propose the methodology as a suitable complement of the set already existing techniques for discovery of functionally significant sequence motifs.

## Additional files:

Additional file 1.
**Table S1**. Comparison of significant pentamer pairs detected with different sequence similarity cutoffs used to eliminate duplicated spacers. Description of data: comparison of significant pentamer pairs detected with sequence similarity cutoffs 65%, 70%, and 75% in three genomes. (DOCX 25 kb)
Additional file 2:
**Table S2.**. Significant motif pairs detected in 569 analyzed genomes. Description of data: The table contains 3326 motif pairs in 569 genomes that identified as significant by our approach. (CSV 300 kb)
Additional file 3:
**Table S3.** Summary statistics for each analyzed genome. Description of data: The table contains mean statistics for variable of interests and distribution of spacer length at the genome level in which at least one significant motif pair is detected. (CSV 31 kb)
Additional file 4:
**Table S4.** List of the 30 genomes selected for detailed analysis. Description of data: The table contains the 30 genomes that are selected to investigate in detail in this paper. (XLSX 10 kb)

## Abbreviations

bp: Base pairs
SD: Shine-Dalgarno
TFBS: Transcription factor binding site
