# Supplementary material for: Unsupervised statistical discovery of spaced motifs in prokaryotic genomes
Source: BMC Genomics. 2017 Jan 5;18:27. doi: 10.1186/s12864-016-3400-0 (PMC5217627; doi:10.1186/s12864-016-3400-0)
Supplement: Additional file 1. — Table S1. Comparison of significant pentamer pairs detected with different sequence similarity cutoffs used to eliminate duplicated spacers. Description of data: comparison of significant pentamer pairs detected with sequence similarity cutoffs 65%, 70%, and 75% in three genomes. (DOCX 25 kb) [file 12864_2016_3400_MOESM1_ESM.docx]

Supplementary Table S1. Comparison of significant pentamer pairs detected with different sequence similarity cutoffs used to eliminate duplicated spacers.

| **Genome** | **Motif pair** |
| --- | --- |
| *Methanopyrus kandleri* AV19 | GTTAT(N)_8_TAACG  CGTTA(N)_10_AACGA  TCGTT(N)_11_AACGA  GTTAT(N)_9_AACGA  CGTTA(N)_9_TAACG  GTTAT(N)_7_ATAAC  CGTTA(N)_7_CGTAA  CGTTA(N)_11_ACGAT  CGAGG(N)_15_TGGAG  TGGAG(N)_17_CGAGG  CGAGG(N)_18_AGGAG  TCGAG(N)_15_CTCGA  AGGAG(N)_15_GAGGA  CTCGA(N)_17_TCGAG  CGAGG(N)_15_TCGAG |
| *Corynebacterium jeikeium* K411 | GGGGA(N)_43_TCCCC  GGGGA(N)_44_CCCCG  GGGGA(N)_45_CCCGC  CGGGG(N)_45_CCCCG  CGGGG(N)_46_CCCGC  GGGGA(N)_56_GGGGA  GGGGA(N)_55_CGGGG  GGGGA(N)_54_GCGGG  CGGGG(N)_55_GCGGG  GCGGG(N)_47_CCCGC |
| *Actinobacillus pleuropneumoniae* L20 | GGTCG(N)_14_TTTGC  CGGTC(N)_16_TTGCA  CGGTC(N)_17_TGCAA  CGGTC(N)_14_TTTTG  AGCGG(N)_19_TGCAA  GCGGT(N)_17_TTGCA  AGCGG(N)_17_TTTGC  GCGGT(N)_16_TTTGC  GGTCA(N)_15_TTGCA  GGTCA(N)_16_TGCAA  GGTCG(N)_15_TTGCA  GGTCG(N)_16_TGCAA  GGTCA(N)_13_TTTTG  GGTCG(N)_13_TTTTG  CGGTC(N)_40_CCGCT  CGGTC(N)_19_CAAAT  CGGTC(N)_15_TTTAC  CGGTC(N)_15_TTTGC  CGGTC(N)_19_CAAAA  CGGTC(N)_18_GCAAA  ACAAG(N)_23_GCAAA  CAAGC(N)_20_TTGCA  CAAGC(N)_21_TGCAA  AGCGG(N)_16_ATTTG  CGGTT(N)_16_TTGCA  CAAGC(N)_19_TTTGC  CAAGC(N)_18_TTTTG  GCGGT(N)_16_TTTAC  CGGTC(N)_13_TTTTT  AGCGG(N)_18_TTGCA  GCGGT(N)_18_TGCAA  TGCAA(N)_19_CGCTT  AGCGG(N)_16_TTTTG  GCGGT(N)_15_TTTTG  GCGGT(N)_19_GCAAA  AAGCG(N)_18_TTTGC  GGTCT(N)_16_TGCAA  GGTCT(N)_15_TTGCA  GGTCT(N)_14_TTTGC  GTCGA(N)_14_TTGCA  GTCGA(N)_15_TGCAA  GTCGA(N)_13_TTTGC  CGGTC(N)_38_GACCG  GGTCA(N)_14_TTTGC  GTCAA(N)_14_TTGCA  AGCGG(N)_19_TACAA  TAAGC(N)_21_TGCAA  GGTCG(N)_17_GCAAA  AGCGG(N)_18_TTACA  GCGGT(N)_18_TACAA  TAAGC(N)_20_TTGCA  TGCAA(N)_21_CTTGT  GCGGT(N)_39_GACCG  TTGCA(N)_22_CTTGT  CGGTC(N)_41_CGCTT  ACAAG(N)_20_TTTGC  TAAGC(N)_19_TTTGC  CAAAA(N)_19_CTTGT  AACAA(N)_22_TTGCA  ATTTG(N)_23_GCTTG  TTGCA(N)_23_TTGTT  TTGCA(N)_17_AACCG  AGCGG(N)_42_CCGCT  AGCGG(N)_17_TTTAC  GCGGT(N)_15_ATTTG  GCGGT(N)_41_CCGCT  AGCGG(N)_21_CAAAT  GCGGT(N)_20_CAAAT  GCGGT(N)_40_ACCGC  TTTGC(N)_22_GCTTG  AGCGG(N)_43_CGCTT  CAAGC(N)_23_CAAAA  AAGCG(N)_18_TTTAC  CGGTT(N)_15_TTTGC  GCGGT(N)_42_CGCTT  AAGCG(N)_22_CAAAT  TTGCA(N)_20_CGCTT  AGCGG(N)_21_CAAAA  AGCGG(N)_20_GCAAA  GCGGT(N)_20_CAAAA  AAGCG(N)_21_GCAAA  AAGCG(N)_17_TTTTG  AAGCG(N)_22_CAAAA  AGCGG(N)_15_ATTTT  AGCGG(N)_15_TTTTT  GCGGT(N)_14_TTTTT |

All pentamer pairs identified as significant with sequence similarity cutoff 75% (as opposed to the 70% cutoff used to generate the bulk of the data in this work) in three genomes are listed. The pairs found also with the 70% and 65% similarity cutoffs are highlighted with magenta background and those found with the 70% cutoff but not with the 65% cutoff are highlighted yellow. Highlighted in green are those pentamer pairs that were not found with the 70% cutoff but are related to the pairs that were found with the 70% cutoff; the relationship involves partial overlap, sharing one of the pentamers, or strong similarity with only one base different. The pentamer pairs found only with the 75% similarity cutoff and unrelated to motifs found with lower cutoffs are highlighted blue. The latter class of significant pentamer pairs was only found in *M. kandleri* and all these pentamer pairs are mutually related and conform to the consensus CTSGAGGAG(N)_12_CTSGAGGA (S stands for G or C). They are found almost exclusively in protein-coding genes and specifically at sites corresponding to imperfect tandem amino acid repeats with biased amino acid composition.
